# Clinical genomics and precision medicine

**DOI:** 10.1590/1678-4685-GMB-2022-0150

**Published:** 2022-10-10

**Authors:** Sérgio D. J. Pena, Eduardo Tarazona-Santos

**Affiliations:** 1 Universidade Federal de Minas Gerais, Instituto de Ciências Biológicas, Departamento de Bioquímica e Imunologia, Belo Horizonte, MG, Brazil.; 2 Núcleo de Genética Médica, Belo Horizonte, MG, Brazil.; 3 Universidade Federal de Minas Gerais, Instituto de Ciências Biológicas, Departamento de Genética, Ecologia e Evolução, Belo Horizonte, MG, Brazil.

**Keywords:** Genomics, whole exome sequence, whole genome sequence, Mendelian disorders, polygenic diseases

## Abstract

Precision Medicine emerges from the genomic paradigm of health and disease. For precise molecular diagnoses of genetic diseases, we must analyze the Whole Exome (WES) or the Whole Genome (WGS). By not needing exon capture, WGS is more powerful to detect single nucleotide variants and copy number variants. In healthy individuals, we can observe monogenic highly penetrant variants, which may be causally responsible for diseases, and also susceptibility variants, associated with common polygenic diseases. But there is the major problem of penetrance. Thus, there is the question of whether it is worthwhile to perform WGS in all healthy individuals as a step towards Precision Medicine. The genetic architecture of disease is consistent with the fact that they are all polygenic. Moreover, ancestry adds another layer of complexity. We are now capable of obtaining Polygenic Risk Scores for all complex diseases using only data from new generation sequencing. Yet, review of available evidence does not at present favor the idea that WGS analyses are sufficiently developed to allow reliable predictions of the risk components for monogenic and polygenic hereditary diseases in healthy individuals. Probably, it is still better for WGS to remain reserved for the diagnosis of pathogenic variants of Mendelian diseases.

## Scientific Medicine and Precision Medicine

William Osler (1849-1919) was a Canadian physician who is considered the “father of scientific medicine”. He revolutionized the teaching of medicine, placing medical education in the university environment, inventing teaching at the patient’s bedside, and creating the system of medical improvement by internship and residency after graduation. He inspired medical studies on the basic sciences, applying the scientific method to clinical medicine, emphasizing that variability in genetic aspects, environmental factors and individual lifestyles modify the evolution and therapeutic response of diseases. One of his famous aphorisms was: “the good doctor treats the disease -- the art of the great doctor is to treat the patient who has the disease.”

In 2015, nearly 100 years after William Osler’s death, Barack Obama, the US President at the time, launched the “Precision Medicine Initiative”. According to him, this was an innovative and ambitious revolution in health and in the treatment of diseases. In his “State of the Nation” speech he explained: “*Doctors have always recognized that every patient is unique, and doctors have always tried to tailor their treatments as best they can to individuals. You can match a blood transfusion to a blood type - that was an important discovery. What if matching a cancer cure to our genetic code was just as easy, just as standard? What if figuring out the right dose of medicine was as simple as taking our temperature?*” (Obama, 2015).

Other names for “ Precision Medicine” are “Personalized Medicine” and “Genomic Medicine”. Physician and molecular biologist Leroy Hood (who received President Obama’s “National Medal of Science”) called it “P4 Medicine” because it is Predictive, Personalized, Preventive, and Participatory at the same time. P4 Medicine focuses on individuals, not populations.

The idea, then, is that underlying human morphological and physiological individuality, there is a genomic individuality. All the physical, intellectual and behavioral characteristics of individuals at a given time are determined by their genome and their life history. Thus, we have the genomic paradigm of health as the harmonious balance between the genome and the environment. The corollary is that disease represents an imbalance between genome and environment.

Precision Medicine naturally emerges from this genomic paradigm of health and disease. Knowing the intimacy of the genomic variations that determine predispositions and resistances, it is possible to manipulate the environment (lifestyle, diet, addition or withdrawal of drugs, preventive surgery, frequency of clinical and laboratory exams) in order to maintain the genome/environment harmonious balance that characterizes health.

## The Human Genome Project and Precision Medicine

Another aphorism of William Osler was: “If it weren’t for the variability among patients, Medicine would be a science and not an art”. This statement brings hope to Precision Medicine, because if there existed a way to control the variability of patients, it would be possible to transform Medicine into a science, i.e., it would be more precise. Knowing that in the human species there is an absolute genomic individuality, one of the ways to achieve this would be with the use of DNA sequencing, as it permits us to know the peculiarities of each person’s genome.

The Human Genome Project (HGM) initiated this possibility (Lander et al., 2001; Venter et al., 2001). It was developed between 1989 and 2003, with the participation of more than five thousand scientists in 250 laboratories and at a cost of 2.7 billion dollars. The result was the sequencing of 99% of the human genome with 99.99% accuracy.

In 2003, at the end of the Human Genome Project, a DNA sequencer could read 50,000 bases a day. As early as 2018, the sequencing equipment was capable of reading 100,000,000 Kb per day, an increase in sequencing capacity of two million times. At the same time, the cost of the automated process of sequencing a person’s genome has decreased, in 2020, to around USD 1,000 (NHGRI, 2020). This faster sequencing was named Next Generation Sequencing (NGS) in honor of the second generation of the *Star Trek TV series.* As NGS depends on an alignment step to the reference human genomic sequence, formally it is a resequencing, not a *de novo* sequencing.

## Next Generation Sequencing

For a complete molecular diagnosis, techniques are needed that analyze the genome as a whole, in a complete and comprehensive way, yet agnostic, that is, independent of initial hypotheses. For pangenomic studies, there are two basic procedures: (1) whole exome sequencing (WES) and (2) whole genome sequencing (WGS). The exome is composed of about 180,000 exons, which are the coding regions for the *circa* 20,000 genes present in the human genome. Although the exome constitutes only 1-2% of the human genome, it is home to 85% of the mutations that cause genetic diseases. Both WGS and WES have the advantage of being unbiased as to the set of genes analyzed, allowing the parallel interrogation of the vast majority of structural genes in the human genome and eliminating the need to predefine genomic targets.

### Whole Exome Sequencing (WES)

Whole exome sequencing (WES), which constitutes a true revolution in medical care, is a diagnostic method to identify molecular defects in patients with suspected genetic disease (Wise et al., 2019; Bick et al., 2019; Costain et al., 2020; Aggarval, 2021; Shickh, 2021).

Traditional medical practice in patients with suspected genetic disease is an attempt to make the diagnosis based on clinical manifestations, imaging tests and biopsies, followed by confirmation by genetic sequencing and screening for candidate gene mutations. Unfortunately, with these procedures, many patients remain without a safe diagnosis, with negative effects, as there are no elements to establish a prognosis, to indicate a specific treatment or to allow genetic counseling of the family. In patients without a definitive diagnosis, it is not uncommon for the family to embark on an exhausting and expensive diagnostic *via crucis* that involves multiple medical consultations, numerous laboratory and imaging tests as well as sequencing of several genes.

Diseases that follow Mendelian patterns of inheritance are known as Mendelian diseases. Approximately 80% of all rare diseases are genetic in origin and most of these diseases are monogenic/Mendelian. It is estimated that they affect at least one person in 50 (Sawyer et al., 2016). 

The total known number of these diseases is estimated to be over 4,500 presently (although it is estimated to exist more than 10,000 of them) and while each is individually rare, together these genetic conditions contribute significantly to morbidity, mortality, and healthcare costs. Estimates suggest that up to 50% of patients with a rare genetic disease never receive a diagnosis. Probably there are 400 million people all over the world suffering from them, In Brazil, the proportion of undiagnosed cases may be even higher, and it can be said that, in our country, they are neglected diseases.

Arriving at an accurate molecular diagnosis of a Mendelian disease has a number of advantages:

- It puts an end to the diagnostic *via crucis;*
- It improves the quality of medical follow-up of the disease, including possible treatments, establishment of prognosis and prevention of complications;
- It allows genetic counseling of families, regarding the risk of recurrence, prenatal diagnosis options and pre-implantation diagnosis;
- It allows the exorcism of parents’ erroneous beliefs and hypotheses about the cause of the disease;
- It allows the parents to achieve emotional closure.

Whole exome sequencing (WES) came to try to resolve cases that remain undiagnosed after other types of detailed and intensive investigations. Evidence from the literature is that WES performed on trios of patients and their parents (three exomic sequencing procedures) allows definitive diagnosis and identification of the genetic defect in 30-50% of patients evaluated for suspected genetic disease. In Brazil, for financial reasons, it is more common for the patient to be tested in isolation, and this is likely to decrease success rates. An economical alternative is to collect DNA samples from the parents and allele-specific validation of only variants in the candidate genes found in their offspring.

In any case, a fundamental diagnostic element is the person who analyzes the variants found in the sequencing. Ideally, it should be done by a professional who has clinical experience in medical genetics and competence in bioinformatics. With this, the same professional can make the best possible assessment of the pathogenicity of the variant(s) found and integrate the results with the clinical picture, to arrive at the correct diagnosis.

Sequencing a person’s exome typically identifies between 35,000 and 40,000 variants. Filtering and prioritizing these variants are the most important, most complex, and most labor-intensive steps. Filtering is essential, since at the beginning there are 35,000-40,000 variants and, among them, only one has to be found, which is the “culprit” for the patient’s illness. In fact, whole exome sequencing should not be seen as a single test, but as millions of tests in which each nucleotide of the exome is identified and pathogenic variants are reported. 

Numerous types of filtering are available, requiring various types of software for a complete evaluation. Among them: (1) genetic filters to suit the mode of inheritance, population frequency of the variant, presence or absence in polymorphism databases; (2) genomic filters focus on reading depth, variant quality and variant effect (change of meaning, absence of meaning, change of reading frame, editing site) and which include *in silico* predictive programs of the possible effect of the variant on proteins and evolutionary conservation, thermodynamic differences and prediction of effect on tertiary structure of proteins; (3) phenotypic filters, which take into account the patient’s clinical characteristics, previous association with the clinical picture and availability in databases.

In this process, all information is important. If the patient is the offspring of a consanguineous couple, for example, it is possible to identify the regions of autozygosity (extended homozygosity) in the genome and peruse with increased attention the variants present in these regions.

Some classes of loss-of-function variants (large deletions, reading window shifts, nonsense mutations, and changes in GT and AG canonical motifs at genomic editing sites) are highly likely to be pathogenic.

Evaluating nucleotide substitutions, which are particularly common, is more difficult. The situation becomes even more complicated when looking for variants associated with autosomal dominant diseases. In these cases, it is useful that the exomes of the patient’s parents are simultaneously sequenced, so that variants that emerge as new mutations can be more easily identified. In general, a *de novo* variant (missing in both parents) is much more likely to be pathogenic than an inherited mutation. 

If the autosomal dominant disorder is familial, the mutation can be seen in other family members. If the mutation does not segregate with the disease, it is highly unlikely that it is implicated in the disease, assuming that penetrance is high. The reverse is not true: co-segregation with disease is not evidence that a variant is pathogenic (a nonpathogenic variant in the disease-associated gene has a 50% chance of residing on the same allele as the true pathogenic mutation, and in this case, will segregate with the disease). A sequence variant found in healthy male and female controls [the gnomAD database (Koch, 2020) contains genome or exome sequencing data from more than 120,000 healthy people from various populations worldwide] would generally be excluded from consideration in a dominant or X-linked condition highly penetrating and with early-onset, but could be pathogenic in an autosomal recessive or low penetrance dominant condition.

In summary, prioritizing variants and classifying their disease-causing capabilities is a challenge. When the probability of the variant being pathogenic is greater than 99%, it is classified as pathogenic*;* if the probability is between 90% and 99%, the variant is called probably pathogenic. If the evidence indicates that the probability of pathogenicity is less than 90% and also does not allow for a confident conclusion that the variant is benign (no health consequences), the variant is called a variant of uncertain significance (VUS). In fact, the expression VUS seems to be poorly understood. Many clinicians seem to interpret VUS as likely benign or non-actionable. This is an error. A VUS may well prove to be pathogenic in the future, once more data are available. A VUS may also be actionable, for example, suggesting new lines of ancillary diagnostic tests.

The ACMG - American College of Medical Genetics and Genomics (Richards et al., 2015) established criteria to classify a given variant into five possibilities: pathogenic, probably pathogenic, of uncertain significance (VUS), probably benign or benign. The use of these criteria is not absolute and is not unambiguous. It is not uncommon to find conflicting classifications of the same variant in the ClinVar database (Landrum et al., 2016). In any case, one cannot forget that pathogenicity is a statement of the probability that the variant is causally related to an inherited disease - it is not a clinical diagnosis. Therefore, it is always recommended to keep in mind that, ultimately, the variant, even if pathogenic, should not be automatically considered to be disease-causing. It needs to be evaluated in the clinical context of the patient, including their phenotype and family history.

### Whole Genome Sequencing (WGS)

In addition to the Whole Exome Sequencing (WES) there is the possibility of Whole Genome Sequencing (WGS) (Belkadi et al., 2015; Lappalainen et al., 2019; Wise et al., 2019; Prokop et al., 2018; Hou *et al*., 2020; Amarasinghe et al., 2020; Nisar et al., 2021).

One challenge of WGS in relation to WES is that, while in WES there is a need to filter 35-40 thousand variants, in WGS we find 5-6 million variants, which makes the study more difficult. With so many variants, a much more precise analysis process is required to find the responsible variant. An example of a tool for this step is the free program Mendel, MD, developed in the laboratory of one of us (Cardenas et al., 2017). Additionally, the size of files grows approximately 10 times, which also complicates the analysis and storage of “raw data”. 

Among geneticists, there has been debate over the use of whole genome sequencing (WGS) versus whole exome sequencing (WES) for the diagnosis of genetic diseases. As the name implies, WGS seeks to sequence the entire genome. Due to the difficulty in sequencing technically challenging regions of the genome with current sequencing platforms (regions with high GC content, repetitive regions, centromeres, telomeres, etc.), the WGS covers about 95% of the genome, although it sequences more than 99.7% of the exons. But there are distinct advantages. For example, exome sequencing (WES) is not able to identify variants when it comes to mutation outside or far from an exon, such as a mutation in the intronic region, in the promoter region of the gene or in the intergenic region, where often are located non-coding DNA variants that may affect gene regulation. Whole genome sequencing (WGS) is able to overcome some of these limitations, as it is capable of diagnosing variants in promoter regions, in other regulatory regions (enhancers*)* and in the middle of introns, although it is necessary for the variants to have already been described previously. This ability to diagnose unknown variants in the non-coding portion of the genome to identify regulatory mutations is still limited, but has continually improved. 

This, however, is not the only reason in favor of the WGS. As already mentioned, WES does not detect pathogenic variants when they are in a gene whose exons are not “fished”, or are fished at low efficiency, by the capture technique, which occurs especially in GC-rich regions. To solve this problem, you can also use WGS, which sequences all exons. By not relying on a capture step, WGS is more powerful than WES in detecting single nucleotide variants and copy number variants, even in regions not well covered by the capture kit (Belkadi et al., 2015). Additionally, WGS is able to detect more CNVs as it covers all breakpoints and detects variants in protein and RNA coding regions that are outside the coverage of the exon capture kit (Belkadi *et al*., 2015; Meienberg et al., 2016). Although currently more expensive, WGS is more powerful than WES for detecting potential disease-causing mutations within WES regions, particularly those due to SNVs.

Currently, WGS costs about twice as much as WES; most of the cost of the WGS corresponds to the sequencing, while the cost of the WES is mainly due to the price of the capture kit. As sequencing costs continue to fall, while the capture kit price still remains stable, there will be a time when the cost of WGS will come closer to WES. Thus, while it is always risky to make projections, it is likely that in the near future the WGS could replace WES in the analysis of human genetic diseases.

## Is it worth doing the complete genomic sequencing of healthy individuals?

**Figure 1 -:**
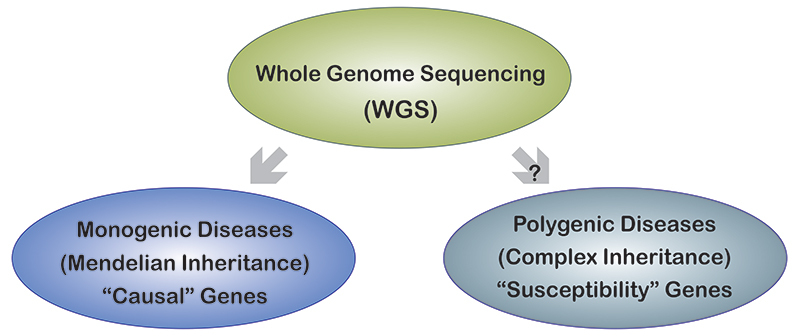
Whole genome sequencing (WGS) has shown its great utility in diagnosing monogenic Mendelian diseases, but it is questionable whether it will be equally useful in accurately assessing the polygenic component of hereditary risk.

In healthy individuals we can observe monogenic highly penetrant variants, which may be causally responsible for existing or future diseases, and also susceptibility variants associated with common polymorphic diseases (see below) with complex inheritance. The question is whether it is worth doing population complete genomic sequencing of healthy individuals to obtain information about these variants (Figure 1).

It was believed that, at least for “monogenic” disorders, genotype-phenotype relationships would be simple and easy to establish, since we were dealing with “causal” genes. But with the advances in genomics, we found that there was a major problem of penetrance, that is, the real probability that an individual who has a pathogenic Mendelian mutation will develop the disease. We can better understand the problem using a true case as an example. In 2019, one of us agreed to carry out in his laboratory the WGS analysis of a healthy Brazilian journalist who wanted to write an article about the process of sequencing his own genome. In the genome analysis, we found in the *VWF gene* (NM_000552.3) the variant c.2561G>A p.(Arg854Gln) which is listed in the ClinVar Database (https://www.ncbi.nlm.nih.gov/ clinvar) as “pathogenic” for types 1 and 2 von Willebrand disease, a clotting disorder. We suggested that he should consult a hematologist and undergo the appropriate biochemical tests. As he reported in his article (Leite, 2019), the Von Willebrand factor dosage in his blood was 85.5%, well within the normal range (50-160%). In this case, the issue was easily resolved because there was an efficient and easily accessible system to test for von Willebrand disease. What if, instead of identifying a variant pathogenic for von Willebrand, I had identified a variant pathogenic for a serious neurological disease, with onset only in old age?

**Figure 2-:**
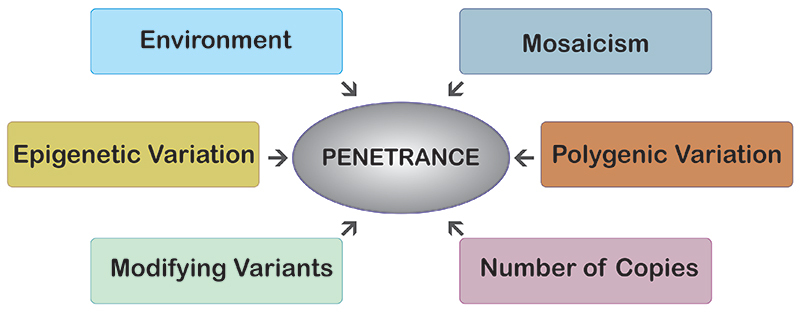
Some factors that may modulate the clinical penetrance of a pathogenic Mendelian variant (modified from Cooper *et al.*, 2013).

But this case is not an exception. Studying 874 genes in 589,306 genomes, Chen et al. (2016) identified 13 adults harboring mutations for eight severe Mendelian conditions, with no clinical manifestations of the indicated disease. This seems to suggest that incomplete penetrance for Mendelian diseases is more common than previously believed. Indeed, incomplete penetrance of well-characterized pathogenic variants for autosomal dominant diseases has been described in a myriad of diseases, including cardiac arrhythmia syndromes (Giudicessi and Ackerman, 2013), hypertrophic cardiomyopathies (Sabater-Molina et al., 2018), immunodeficiencies (Gruber and Bogunovic, 2013) and cancer susceptibility mutations (Tung et al., 2016), just to name a few. Furthermore, incomplete penetrance may explain not only why dominant genetic diseases are occasionally passed on from clinically unaffected parents to their offspring, but also why healthy individuals can harbor large numbers of potentially disadvantageous variants in their genomes without suffering any obvious harmful effects (Cooper et al., 2013). Even without considering the more obvious and trivial “age-related non-penetrance” for late-onset diseases and “sex-related non-penetrance” for gender-specific diseases, incomplete penetrance is still very frequent. After all, Mendelian diseases involve a single gene, while there are about 20,000 of them in the human genome, which creates many opportunities for a genetic or epigenetic modification of the phenotypic effects of the pathogenic mutant allele (Figure 2). Environmental factors can also play a role. Also, very recently, Kingdom et al. (2022) have shown that many genes routinely tested within pediatric genetics have deleterious variants with intermediate penetrance that may cause lifelong sub-clinical phenotypes in the general population.

We do not want to go into great detail here and the reader looking for more complete information should refer to the rather thorough review by Cooper et al. (2013), from which we obtained the inspiration for Figure 2. Thus, it no longer seems formally appropriate to consider Mendelian diseases as simply monogenic *sensu strictu* (Cooper *et al*., 2013). In other words, incomplete penetrance arising from the complex interaction between the superabundance of genetic variation present in the human genome, coupled with environmental factors, is likely to occur for all diseases (Gruber and Bogunovic, 2020). Of course, the pathogenic variant may be monogenic, but its disease expression is multifactorial, probably often with a polygenic component. As pointed out by Visscher et al. (2021), the genetic architecture of disease, whether rare or common, is consistent with the hypothesis that all diseases are polygenic.

## Ancestry and Precision Medicine

Ancestry adds an additional layer of complexity for the future of precision medicine, involving both Mendelian and complex diseases, particularly in admixed populations such as Brazilians. Due to their history of admixture, genomes of Brazilians are mosaics of fragments with different origins (mostly European, African and Native American). However, because populations with European ancestry are the most studied, we know much more about the mutations that cause diseases in individuals of European origin. For instance, loss-of-function mutations, if placed in fragments of our genomes that are of African and Native American origins, are three times less likely to be present in the ClinVar dataset than loss-of-function mutations placed in fragments of European origins (Naslavsky et al., 2021). While it is not clear yet how the effects of ancestry should be incorporated into clinics (including the Polygenic Risk Scores, see below), there are at least three cases in which ancestry is relevant: (i) mutations causative of rare genetic variants may be associated with some ancestries. For instance, for cystic fibrosis, that presents allelic heterogeneity, mutation ΔF508, which is largely the most common in Europe, is not necessarily the most common in Sub-Saharan Africa. In South African blacks, the most common mutation of cystic fibrosis in many populations is different (3120+1G->A, Padoa et al., 1999); (ii) the same SNV may have different effect sizes in individuals with different levels of ancestry. For instance, variant rs2836365 in the ERG gene, which is a susceptibility SNV for acute lymphoblastic leukemia, has higher odd ratio in individuals with more Native American ancestry (Qian et al., 2019); (iii) in some instances, even the origin of the piece of the genome where genetic variants are located may be relevant (*i.e.*, the local chromosome ancestry). For example, in Latin American lung cancer patients, somatic mutations in the EFGR gene (that lead to a molecular characterization of the tumor) are more likely to occur in fragments of the genome of Native American origin (Carrot-Zhang et al., 2021). About 60% of non-small cell lung carcinomas (NSCLCs) express EGFR, which has become a therapeutic target for the treatment of these tumors. 

## Complex Inheritance Diseases (Polygenic Diseases)

In the last 15 years, genome-wide association studies (GWAS) have contributed to the identification of the relation of specific genomic regions with an impressive number of common diseases with complex inheritance, including breast cancer, ovarian cancer, colorectal cancer, arterial disease, coronary heart disease, type 2 diabetes, autoimmune diseases, psychoses, etc. As of September 2018, the NHGRI-EBI catalog of such studies contained 5,687 GWAS comprising 71,673 associations of genetic variants with phenotypic characteristics from 3,567 publications (Buniello et al., 2019).

The Polygenic Risk Score (PRS) for a given disease of complex inheritance is an individual risk index that proposes to summarize the aggregate effect of hundreds to thousands of genetic variants from the entire human genome into a single number (Lambert et al., 2019; Ala-Korpela and Holmes, 2020). For this, the number of susceptibility alleles carried by an individual in all typed single nucleotide polymorphisms (SNP) in an individual’s genome is used, weighted by their magnitudes of the effect between the genotype in a given SNP and the complex inheritance disease of interest. There are several methods for calculating polygenic risk scores, ranging from including only SNPs that have exceeded genome-wide significance thresholds to the more modern use of millions of SNPs encompassing all those that individually associate, even if very weakly, with the phenotype of interest. 

Polygenic risk scores (PRSs) aggregate the many small effects of alleles across the human genome to estimate the risk of a disease or disease-related trait for an individual. Potential benefits of PRSs include cost-effective improvement of primary disease prevention, more refined diagnoses and greater accuracy in drug prescribing. However, these must be weighed against potential risks such as uncertainties and biases in PRS performance, as well as potential misunderstandings and misuse of these in medical practice and society at large. While not a diagnosis in itself, PRSs generally provide information that can be used to enhance or guide, but not replace, risk prediction models and diagnostic pathways. In essence, in addition to being based on an individual’s germline genome, a PRS can be treated like any other risk predictor. A single genetic test per individual provides raw genetic information that can be used to generate many polygenic risk scores (for heart disease, diabetes, breast cancer, etc.) from existing genetic data. However, it should be noted that providing PRS based on common variants, but not considering or testing high-impact rare variants, may provide a substantially incomplete risk estimate for individuals, especially those with a family history (Polygenic Risk Score Task Force of the International Common Disease Alliance, 2021).

More recently, there have been proposals that the “standard” method of calculating polygenic hazards by genome-wide association studies (GWAS) followed by imputation can be advantageously replaced by whole genome sequencing (Homburger et al., 2019). Indeed, Khera et al. (2019) showed in detail how to obtain a Polygenic Risk Score using only next-generation sequencing (NGS) data. For clinicians, the promise of the Polygenic Risk Score method for estimating the risk of a disease with a complex inheritance (polygenic disease) is very attractive. 

The same happened with many other diseases with complex inheritance, as can be easily verified through a search on a published catalog (Polygenic Score Catalog, 2022), an open resource of scores (including variants, alleles and weights) and consistently curated metadata required for reproducibility and independent applications (Lambert et al., 2021).

Yet, serious doubts about the predictive value of polygenic risk scores have arisen, both from an experimental and a theoretical point of view. Experimentally, some careful studies have failed to confirm the clinical utility and clinical validity of polygenic risk scores. For example, Mosley et al. (2020) performed a retrospective cohort study that included 7,237 middle-aged participants of European descent free of clinical coronary heart disease at baseline. When they added a polygenic risk score to the American College of Cardiology and American Heart Association 2013 pooled cohort equations, it did not significantly improve risk discrimination, calibration, or reclassification compared to conventional predictors. They concluded that a polygenic risk score may not be able to increase the risk prediction in the general middle-aged population. Likewise, Lewis and Vassos (2020) noted that the association between polygenic risk scores and disease status may have been confirmed in investigational studies, but that its clinical utility has not yet been demonstrated.

In the context of ancestry and admixture, the transferability of Polygenic Risks Scores (PRS) between populations is also critical. Most PRS have been developed and validated in populations with European ancestry, and their predictive values seem to be lower in individuals of non-European or mixed ancestry (Bitarello and Mathieson 2020, Ruan et al. 2022). This is at least partially due to the uncertainty in incorporating non-European susceptibility variants (that may be simply unknown), as well as their effect sizes. In the framework of Latin America, from the more than 2,000 PRSs present in the PSG Catalog in March 2022, only few dozens have been validated in Latin American/US Hispanics populations. 

Perhaps the most severe criticism has come from theoretical grounds. In a very compelling article, Wald and Old (2019) noted that the hope that individuals identified by high polygenic risk scores might benefit from preventive interventions rests on the incorrect assumption that odds ratios derived from polygenic risk scores are directly useful for population screening and disease risk prediction. The authors point out that estimates of the relative risk between a disease marker and a disease need to be extremely high for the risk factor to merit consideration as a valid screening test. According to them, we should avoid unrealistic expectations regarding our medical exams (Wald and Old, 2019). 

The most prudent attitude at the moment seems to be conservative. We should avoid over-optimism and not make medical use of polygenic risk scores until new studies and publications definitively establish their clinical utility and clinical validity. In conclusion, the review of available evidence does not favor the idea that, at this time, whole genome sequencing (WGS) is sufficiently developed to allow reliable predictions of monogenic and polygenic components of hereditary disease risk in healthy individuals. Probably it is better that WGS is still reserved for the diagnosis of pathogenic variants of Mendelian diseases.
